# Atrial Fibrillation in Athletes: Mechanisms, Management, and Future Directions

**DOI:** 10.1016/j.jaccas.2026.107061

**Published:** 2026-03-25

**Authors:** Ibrahim Alameh, Ghina Al Fout, Michael D. Ezekowitz, Mohammed Kamareddine

**Affiliations:** a Department of Internal Medicine, Lankenau Medical Center, Wynnewood, Pennsylvania, USA; b Department of Internal Medicine, Temple University Hospital, Philadelphia, Pennsylvania, USA; c Sydney Kimmel Medical School, Thomas Jefferson University, Philadelphia, Pennsylvania, USA; d Department of Cardiology, Lankenau Heart Institute, Bryn Mawr Hospital/Mainline Health, Wynnewood, Pennsylvania, USA; e Department of Cardiology, Lankenau Medical Center, Wynnewood, Pennsylvania, USA

**Keywords:** ablation, anticoagulation, atrial fibrillation, electrophysiology, palpitations

## Abstract

**Background:**

High-volume endurance training may shift exercise from cardioprotective to arrhythmogenic, increasing the risk of atrial fibrillation (AF) in healthy athletes.

**Case Summary:**

A 38-year-old marathon runner with a 15-year history of high-intensity endurance training presented with episodic palpitations and reduced athletic performance. Electrocardiogram showed paroxysmal AF; echocardiography revealed mild left atrial enlargement without structural heart disease. Symptoms worsened during the recovery phase after runs, suggesting vagally mediated AF. After unsuccessful flecainide therapy, he underwent pulmonary vein isolation. Twelve months later, he remained free of recurrent AF on Holter monitoring and successfully resumed endurance training following an 8-week recovery period. However, ongoing high-volume exercise may still carry a residual risk of arrhythmia.

**Review Summary:**

AF in athletes is associated with a 2- to 5-fold increased prevalence linked to atrial remodeling, autonomic imbalance, and inflammation. Rhythm control, especially catheter ablation, preserves performance and quality of life.

**Take-Home Message:**

Excessive endurance training increases AF risk; early rhythm control and structured return-to-play strategies optimize outcomes.

## Graphical Abstract

**Figure:**
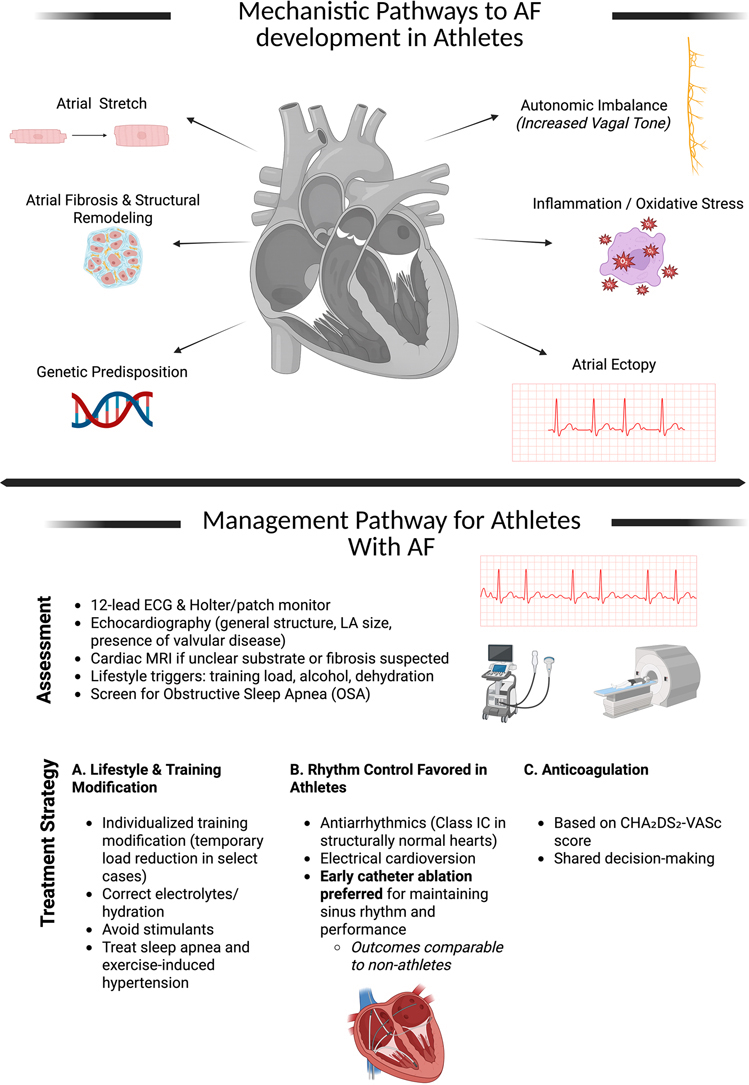

Regular physical activity is among the most effective lifestyle interventions for promoting cardiovascular health.^1^ Its contributions to reduced mortality, atherosclerosis, and complications of hypertension and diabetes have been well established; however, some paradoxes emerge.^2^ Dyslipidemia and exercise often coexist, creating a complex interplay in which acute exercise has been shown to transiently increase arterial wall permeability, potentially facilitating focal deposition of circulating proteins, including lipoproteins, within the arterial wall, leading to lipid elevation and atherosclerosis.^3^^,^^4^ Another paradoxical phenomenon is the relationship between endurance training and the likelihood of developing atrial fibrillation (AF). There is evidence that endurance training increases, rather than decreases, the likelihood of developing AF in young athletes without underlying cardiac disease.^5^ This condition, known as athlete's AF, has been recognized for several decades and is well documented in epidemiologic studies.^5^, ^6^, ^7^ The 2023 guidelines reported by the American College of Cardiology, the American Heart Association, the American College of Chest Physicians, and Heart Rhythm Society acknowledge that athletes engaging in sustained, high-volume endurance exercise, typically exceeding 45 metabolic equivalent hours per week, exhibit a higher incidence of AF compared with their sedentary counterparts.^8^^,^^9^ Conversely, engaging in moderate-intensity exercise offers protective benefits against AF, resulting in a J-shaped relationship between exercise volume and risk.^10^^,^^11^

**Take-Home Messages**

• Excessive lifelong endurance training increases the risk of AF through atrial remodeling, autonomic imbalance, and inflammation.
• Rhythm control, particularly catheter ablation, is preferred over rate control therapy in athletes, as it preserves athletic performance and facilitates a safe return to competitive sport.

Athletes with AF often present unique diagnostic and therapeutic challenges. They are typically younger, are more physically active, and usually have structurally normal hearts aside from possible mild atrial remodeling. Although AF in athletes is increasingly recognized and frequently encountered in tertiary arrhythmia centers, evaluation and management remain distinct from evaluation and management of typical patients with AFs. Because athletic identity and competitive performance are central to their well-being, management decisions in these patients must go beyond arrhythmia suppression to include quality of life, sport participation, and performance maintenance. This article uses the case of an athlete with AF to highlight athlete-specific considerations and to illustrate management strategies, with the goal of returning to the pre-AF clinical condition as the primary end point.

## Mini-Review

### Epidemiology and risk in athletes

Multiple meta-analyses and cohort studies have confirmed a 2- to 5-fold higher prevalence of AF among endurance athletes compared with age- and sex-matched control patients.^5^^,^^12^ The association between long-term endurance sport participation and AF was first described more than 2 decades ago by Mont et al,^13^ establishing the foundation for the concept of athlete's AF. In later large-scale investigation, Andersen et al^6^ studied more than 52,000 cross-country skiers. They demonstrated a stepwise increase in AF risk with greater race participation and faster performance times. This finding established a dose-response relationship between cumulative exercise exposure and arrhythmia incidence. Similar patterns have been reported among cyclists, runners, and triathletes.^14^, ^15^, ^16^

The risk is most pronounced among athletes engaged in high-intensity, long-duration endurance activities such as marathon running, rowing, and cross-country skiing, particularly in men older than 40 years of age.^6^^,^^17^^,^^18^ Observational data support a U- or J-shaped relationship: moderate physical activity is protective, whereas long-term high-volume, high-intensity training increases AF risk, with commonly cited exposure thresholds of roughly 1,500 to 2,000 cumulative hours of intense endurance training.^7^^,^^19^, ^20^, ^21^

Non–exercise-related risk modifiers common in athletes, such as sleep apnea, alcohol consumption (holiday heart syndrome), and dehydration, further amplify susceptibility.^22^^,^^23^ Importantly, AF in female athletes is increasingly recognized, though data remain limited.^24^ Although male sex is an independent risk factor, emerging studies suggest that postmenopausal female athletes who engage in lifelong endurance training may also exhibit elevated risk compared with sedentary women, warranting sex-specific investigation.^12^

In clinical practice, AF in athletes most often manifests as paroxysmal and vagally mediated episodes occurring at rest, during recovery, or nocturnally. The arrhythmia burden varies widely, from asymptomatic episodes detected on wearable monitors to symptomatic, performance-limiting palpitations or exercise intolerance.^15^^,^^25^^,^^26^

### Pathophysiology

**Figure 1:**
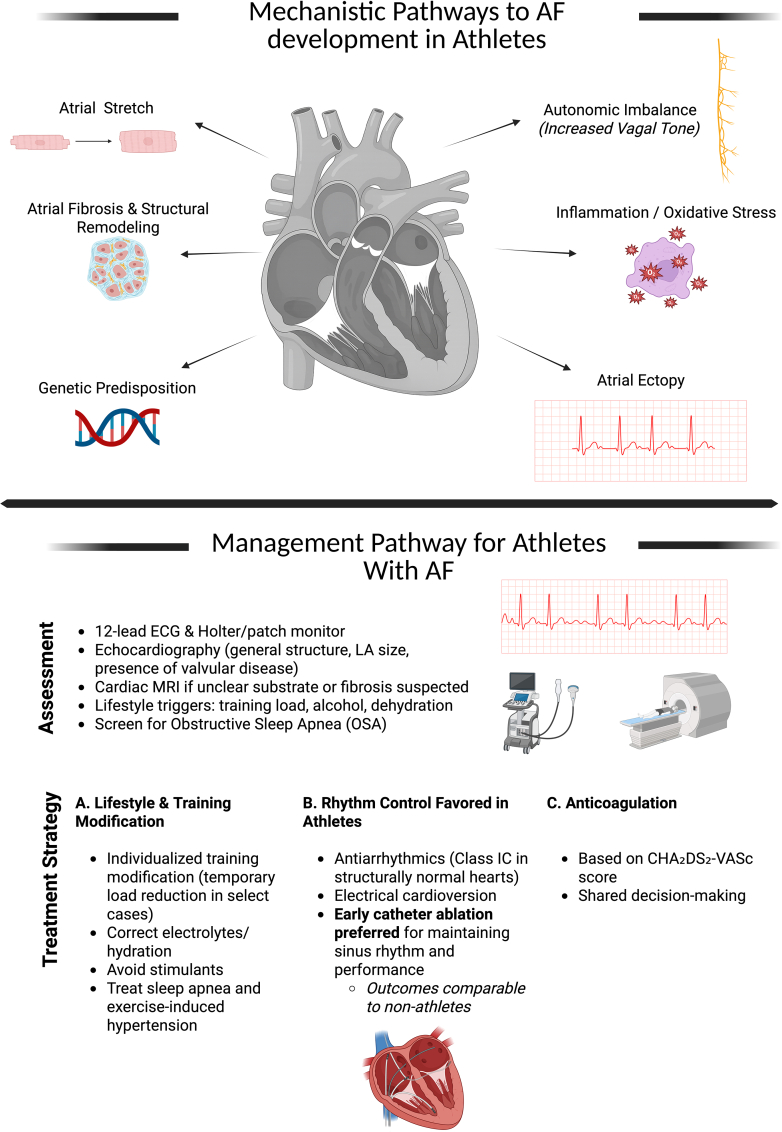
Integrated Mechanistic and Therapeutic Framework for Atrial Fibrillation in Athletes Created in BioRender. Alameh, I. (2026). https://BioRender.com/bt6dc55. AF = atrial fibrillation; ECG = electrocardiogram; MRI = magnetic resonance imaging.

The mechanisms of AF in athletes are multifactorial, involving the interplay of structural, electrical, autonomic, and inflammatory pathways (Figure 1).^23^^,^^27^ Chronic endurance training imposes repeated volume and pressure overload on the atria, leading to atrial dilation, increased wall stress, and progressive remodeling. Left atrial enlargement, often accompanied by right atrial dilation, is common among endurance athletes and correlates with training intensity and duration. This structural adaptation may initially be physiological, facilitating stroke volume and diastolic filling, but prolonged exposure can promote fibrosis and conduction heterogeneity, creating a substrate for re-entry.^28^^,^^29^

In addition to atrial dilation, animal endurance models have demonstrated progressive atrial hypertrophy, fibrosis, and vagal-dependent AF vulnerability. Imaging studies suggest that cardiac magnetic resonance imaging (MRI) can detect left atrial fibrosis with late gadolinium enhancement.^15^^,^^30^

Autonomic imbalance represents another distinctive hallmark of athlete AF. Endurance training induces high resting vagal tone and enhanced parasympathetic dominance, shortening the atrial effective refractory period and facilitating triggered activity. During exercise, sympathetic surge and catecholamine release can generate focal ectopy, particularly in the pulmonary veins. The alternation between increased vagal tone at rest and sympathetic activation during training amplifies the risk of AF initiation in a structurally remodeled atrium.^6^^,^^31^^,^^32^

Inflammatory and oxidative pathways further contribute to the arrhythmogenic milieu. Repeated bouts of strenuous exercise can cause transient myocardial injury, reflected by elevated troponin levels and C-reactive protein, and may trigger low-grade inflammation and fibrosis.^33^^,^^34^

Finally, genetic predisposition may modulate susceptibility to AF, with rare loss-of-function variants in genes such as *TTN*, which is associated with early-onset AF.^35^ Emerging evidence indicates that polygenic AF risk significantly modifies arrhythmia susceptibility in endurance athletes exposed to high cumulative exercise, highlighting the importance of gene-environment interactions in AF development.^36^

### Management in athletes

Management of AF in athletes focuses on aggressive arrhythmia suppression, performance preservation, and safety assurance (Figure 1).

#### Lifestyle and exercise modification

For athletes with paroxysmal AF, initial management should include addressing modifiable factors: reducing alcohol intake, optimizing blood pressure control, treating sleep apnea, and regulating excessive training loads.^37^ Although it has been reported that some athletes experience fewer AF episodes when they substantially reduce high-intensity endurance training, there is no evidence that detraining periods decrease AF recurrence or improve rhythm stability.^38^ The role of structured detraining remains controversial. Prospective data demonstrating sustained rhythm control or atrial reverse remodeling are limited. Consequently, detraining should be individualized and balanced against psychological well-being, competitive goals, and overall cardiovascular fitness.^39^^,^^40^

#### Rhythm vs rate control

For symptomatic athletes, rhythm control is preferred to rate control; the latter is often poorly tolerated because beta blockers and certain calcium channel blockers reduce maximal heart rate and impair exercise performance. Antiarrhythmic drugs (AADs) such as class Ic antiarrhythmics (flecainide, propafenone) may be considered in athletes without structural heart disease. However, long-term adherence is low because of side effects and doping restrictions.^41^ Amiodarone, though effective, is avoided in young or competitive athletes because of toxicity and performance implications.^23^

#### Catheter ablation

Catheter ablation, typically pulmonary vein isolation, has become the cornerstone of rhythm control for athletes with symptomatic or recurrent AF.^42^ Procedural efficacy in athletes is comparable to that of nonathletic patients, with success rates exceeding 80% after single or repeat ablations, suggesting that high-level athletic activity does not adversely affect ablation efficacy.^42^ In multiple series, including large prospective registries, freedom from AF at 1 year approached 86% for paroxysmal AF and 68% for persistent AF, with sustained improvements in quality of life and exercise tolerance.^42^ In addition, pulmonary vein isolation significantly reduced the use of AADs and rate-controlling agents, with only 14.8% of athletes using AADs and 28.4% using rate-controlling agents at the last follow-up (*P* < 0.001).^42^

Predictors of ablation success include paroxysmal presentation, absence of significant atrial fibrosis, and shorter AF duration before intervention.^42^ Persistent AF, advanced atrial myopathy, and delayed ablation are associated with higher recurrence rates. Return-to-play after ablation typically involves a short restriction (about 1-2 weeks) followed by a graded return.^43^ Importantly, returning to competitive sport after successful ablation is feasible, with most athletes resuming training within 2 to 3 months post-procedure.^44^^,^^45^

#### Anticoagulation

Thromboembolic risk assessment for athlete’s AF follows standard CHA_2_DS_2_-VASc criteria, although most endurance athletes fall into low-risk categories in which long-term anticoagulation is not required, as in the present case. However, for athletes requiring therapy, direct oral anticoagulants are preferred over warfarin for convenience and safety.^8^^,^^46^, ^47^, ^48^, ^49^ Contact and collision sports pose additional bleeding hazards, necessitating individualized risk-benefit discussions.^15^ Emerging strategies incorporating AF burden assessment via continuous monitoring and intermittent or on-demand anticoagulation are under investigation.

#### Shared decision-making and multidisciplinary care

Management of AF in athletes mandates shared decision making involving the athlete, electrophysiologist, sports cardiologist, and team physician. Discussions should encompass arrhythmia control options, eligibility for sport, antidoping implications, and long-term cardiovascular health. For competitive athletes, collaboration with governing sports bodies ensures compliance with participation regulations and safety standards.

### Athlete-specific considerations

Athletes differ from typical patients with AF in presentation, priorities, and therapeutic thresholds. Many athletes are asymptomatic, with arrhythmia detected incidentally through wearable devices or precompetition screening. Others experience subtle declines in performance rather than overt palpitations.^15^^,^^25^ The psychological impact of AF, fear of recurrence or of using anticoagulation, loss of control, or disqualification from competition can be substantial and warrants counseling support.

Diagnostic evaluation should include resting and exercise echocardiography, electrocardiogram, and often cardiac MRI to quantify atrial size, detect fibrosis, and exclude structural disease.^15^^,^^50^ Exercise testing is important as it assesses rate response and uncovers exercise-induced ectopy or arrhythmia.

Following ablation, resting sinus heart rate may increase modestly (approximately 10 beats/min) owing to reduced vagal tone; however, the clinical significance of this change varies, and its impact on endurance performance remains incompletely defined.^51^ Repeat ablation is occasionally necessary, particularly for atypical atrial flutter or pulmonary vein reconnection. Ablation strategy should avoid unnecessary lesion sets that risk stiff left atrial syndrome, which can reduce cardiac output during exertion.^52^

Eligibility for return to competitive sports depends on arrhythmia control, left ventricular function, and anticoagulation status. According to the 2020 European Society of Cardiology consensus, athletes free of symptomatic AF and with a controlled ventricular response can resume competition after at least 3 months of stable rhythm.^53^ Ongoing rhythm monitoring with electrocardiogram patches or smart devices facilitates early detection of recurrence. Despite successful rhythm control, ongoing exposure to high-volume endurance training may continue to sustain the underlying arrhythmogenic substrate.

### Synthesis

Endurance athletes constitute a distinct phenotype of AF pathophysiology. Chronic repetitive hemodynamic stress, atrial remodeling, and autonomic adaptation generate a proarrhythmic substrate even in the absence of traditional cardiovascular risk factors. The resulting arrhythmia, though often benign, carries implications for quality of life, performance, and long-term atrial function.

Management should be individualized, integrating lifestyle modification, early rhythm control (often via ablation), and structured follow-up. Importantly, the athlete's identity, motivation, and goals must be central to all decisions. The convergence of cardiology, sports medicine, and electrophysiology has created a new subspecialty, sports electrophysiology, dedicated to balancing arrhythmia suppression with the preservation of athletic performance.

## Future Directions

Future research on athlete's AF should refine risk stratification by identifying the thresholds of exercise intensity and cumulative lifetime exposure at which the physiological benefits of exercise shift toward arrhythmogenic remodeling. This includes investigating sex-based differences and genetic susceptibility to explain why some athletes develop AF despite similar training volumes.

Advances in imaging and molecular diagnostics, such as atrial strain analysis, late gadolinium enhancement MRI, and biomarker or microRNA profiling, may be crucial for early detection by revealing subclinical atrial remodeling before arrhythmia occurs. Prospective studies should also evaluate the effects of structured detraining, cross-training, or adjusted exercise intensity to establish safe and effective exercise prescriptions for athletes with AF.

Additionally, multicenter registries should track long-term ablation outcomes, recurrence rates, and sports performance to strengthen data-driven return-to-play guidelines. Ultimately, developing standardized frameworks that integrate arrhythmia burden, anticoagulation management, and sport-specific trauma risks will be crucial for ensuring both safety and continued athletic participation.

## Conclusions

High-volume endurance exercise increases the AF risk in athletes, highlighting the balance between beneficial cardiac adaptation and maladaptive remodeling. Whereas moderate activity remains protective, chronic extreme training can cause atrial fibrosis, dilation, and autonomic changes predisposing to arrhythmia. Catheter ablation offers effective rhythm control with excellent long-term success and preserved exercise capacity in selected athletes. Individualized management, using shared decision making, tailored exercise prescription, and integration of electrophysiological and sports cardiology expertise, optimizes outcomes and helps athletes maintain both cardiovascular health and competitive identity.

## Funding Support and Author Disclosures

Funding from the Vasiliou Family was used to support the publication fees associated with this article. Dr Ezekowitz reports consulting relationships with Bristol Myers Squibb, Daiichi Sankyo, Medtronic, Johnson & Johnson, and Boston Scientific. He has served as principal investigator or co-principal investigator for the SPINAF, PETRO, RE-LY, RE-LYABLE, EXPLORE-Xa, X-VERT, and EMANATE trials. He has served on the executive committees of the ENGAGE AF-TIMI 48 and ENSURE-AF trials. He is a former member of the atrial fibrillation guideline writing committee. All other authors have reported that they have no relationships relevant to the contents of this paper to disclose.
